# Effectiveness of a web-based computer-tailored intervention promoting physical activity for adults from Quebec City: a randomized controlled trial

**DOI:** 10.1080/21642850.2020.1850287

**Published:** 2020-12-08

**Authors:** François Boudreau, Gilles R. Dagenais, Hein de Vries, Michel Jean Louis Walthouwer, José Côté, Ginette Turbide, Anne-Sophie Bourlaud, Paul Poirier

**Affiliations:** a Département des sciences infirmières, Université du Québec à Trois-Rivières, Trois-Rivières, Canada; b Institut Universitaire de Cardiologie et de Pneumologie de Québec, Québec, Canada; c Département de médecine, Faculté de médecine, Université Laval, Québec, Canada; d School for Public Health and Primary Care (CAPHRI), Department of Health Promotion, Maastricht University, Maastricht, Netherlands; e Faculté des sciences infirmières, Centre de recherche du Centre hospitalier de l’Université de Montréal, Quebec, Canada; f Faculté de Pharmacie, Université Laval, Québec, Canada

**Keywords:** Physical activity, web-based, computer tailoring, adults

## Abstract

**Background:**

The primary objective of this study was to determine the effectiveness of a 3-month web-based computer-tailored intervention on moderate-to-vigorous physical activity (MVPA) in adults.

**Methods:**

A total of 242 Canadian adults aged between 35 and 70 years were randomized to an experimental group receiving the intervention or a waiting list control group. The fully automated web-based computer-tailored physical activity intervention consists of seven 10- to 15-min sessions over an 8-week period. The theoretical underpinning of the intervention is based on the I-Change Model.

**Results:**

A repeated-measures ANOVA using a linear mixed model showed a significant ‘group-by-time’ interaction favoring the intervention group in self-reported MVPA (*p* = .02). The MVPA was similar in both groups at baseline (mean ± SD; 176 ± 13 vs. 172 ± 15 min/week, *p* = .72) and higher in the intervention than in the control group at a 3-month follow-up (259 ± 21 vs. 201 ± 22 min/week, *p* = .04). This finding was comparable across women and men (group-by-sex, *p* = .57) and across participants meeting or not physical activity guidelines at baseline (group-by-baseline physical activity, *p *= .43). Although engagement to the web-based sessions declined over time, participants completing more web sessions achieved higher self-reported MVPA (*p* < .05).

**Conclusion:**

These findings suggest that this intervention is effective in enhancing self-reported MVPA in this adult population in the short term; however, this needs to be confirmed in a larger trial with better engagement to the web-based sessions.

In the field of behavioral medicine, more behavior change interventions are moving from the traditional patient–provider encounters to eHealth technology platforms (Michie, Yardley, West, Patrick, & Greaves, 2017; Moller et al., 2017; Webb, Joseph, Yardley, & Michie, 2010). Although the scope of definitions of eHealth has varied considerably over the past 15 years (Boogerd, Arts, Engelen, & van de Belt, 2015; Oh, Rizo, Enkin, & Jadad, 2005; Shaw et al., 2017), researchers, policymakers, and practitioners nevertheless agree that these technologies represent a promising public health low-cost approach. Most especially, eHealth technology can simultaneously reach a large number of a population target group (e.g. adults) to promote and maintain their health through primary or secondary prevention and management of health problems (Bennett & Glasgow, 2009). Among the eHealth technologies available are those dedicated to supporting healthy lifestyle behaviors (Shaw et al., 2017). In this regard, the promotion of regular physical activity (PA) has received significant research attention over the last 20 years (Müller et al., 2018). While many systematic reviews reveal the effectiveness of eHealth technologies in making more people physically active (Cotie et al., 2018; Davies, Spence, Vandelanotte, Caperchione, & Mummery, 2012; Jahangiry, Farhangi, Shab-Bidar, Rezaei, & Pashaei, 2017; LaPlante & Peng, 2011), levels of insufficient physical activity are particularly high and still rising in high-income countries (Guthold, Stevens, Riley, & Bull, 2018). For instance, in Canada, this behavior poses a public health challenge, as only 15% of Canadian adults achieve the recommended 150 min of moderate-to-vigorous physical activity (MVPA) weekly (Statistics Canada, 2015).

Among eHealth technologies, computer-tailoring technology is one of the most widely used to promote regular physical activity (systematic reviews (Broekhuizen, Kroeze, van Poppel, Oenema, & Brug, 2012; Neville, O’Hara, & Milat, 2009; Short, James, Plotnikoff, & Girgis, 2011) and meta-analyses (Krebs, Prochaska, & Rossi, 2010; Noar, Benac, & Harris, 2007)). This technology provides an innovative and potentially beneficial avenue to reach many of the insufficiently active Canadian adults to increase their levels of MVPA. This technology assesses individuals’ perceived health behavior status and the determinants of their motivation and behavior. Using data-driven decision rules, the technology provides automated tailored feedback about these determinants based on participants’ answers and personal characteristics (de Vries & Brug, 1999; Krebs et al., 2010). The rationale underpinning computer-tailoring technology is based on the Elaboration Likelihood Model (Hawkins, Kreuter, Resnicow, Fishbein, & Dijkstra, 2008). In brief, by enhancing the perceived relevance of health messages and thus personal involvement, as shown by studies using functional magnetic resonance imaging (Chua, Polk, Welsh, Liberzon, & Strecher, 2009; Chua et al., 2011), computer-tailoring technology enables users to give more thoughtful consideration of health messages. Hence, using the central route of information processing will determine the extent of cognition changes (e.g. attitude, self-efficacy) and then support the adoption of healthy lifestyle behaviors. In addition to its automated yet personalized nature (de Vries & Brug, 1999), several advantages are associated with computer-tailoring (Lustria et al., 2013). These advantages include the possibility of interacting with many users under free-living conditions (Schulz, Kremers, et al., 2014) as well as its cost-effectiveness (Peels et al., 2014; Schulz, Smit, et al., 2014) and cost-utility (Schulz, Smit, et al., 2014).

While computer-tailored interventions have the potential to favorably influence health behaviors (Krebs et al., 2010; Lustria et al., 2013), more research is needed regarding some issues pertaining to physical activity (PA). Davies et al. (2012) meta-analysis of internet-delivered PA interventions, including computer-tailoring technology, showed that studies classifying participants as less physically active (e.g. not reaching PA guidelines) at baseline produced greater effects on PA behavior than studies that did not screen participants for baseline PA levels. Yet, most new research on computer-tailored PA interventions has analyzed the interventions’ effectiveness without considering the participants’ baseline PA levels (Friederichs, Oenema, Bolman, & Lechner, 2015; Golsteijn et al., 2018; Gomez Quiñonez, Walthouwer, Schulz, & de Vries, 2016; Peels et al., 2014; Plaete, De Bourdeaudhuij, Verloigne, & Crombez, 2015). This suggests that future computer-tailored intervention studies should assess adults who do not meet PA guidelines when measuring their effectiveness.

Achieving the recommended 150 min per week of MVPA is apparently more challenging for Canadian women than it is for Canadian men (Colley et al., 2011), perhaps due to men’s and women’s differing beliefs and motives to participate in MVPA (Hankonen, Absetz, Ghisletta, Renner, & Uutela, 2010; Koivula, 1999; Molanorouzi, Khoo, & Morris, 2015; Rhodes et al., 2014). This suggests that specific beliefs and motives may need to be integrated when designing an intervention in order to promote MVPA among adult women (Edwards & Sackett, 2016). Computer-tailoring technology offers precisely this possibility by developing customized messages to promote women’s regular participation in MVPA. Another issue concerns participants’ education levels. Higher attrition rates among participants with low- or mid-level education have been documented in computer-tailored interventions, possibly due to difficulties processing information (Reinwand et al., 2015). Relative to text-delivery tailored feedback, video-delivery tailored feedback has been found to be more efficient at gaining participants’ visual attention in a web-based PA intervention (Alley et al., 2014); however, this last observation was analyzed without assessment of the participants’ educational attainment. There is a need to determine whether video-delivery tailored PA feedback facilitates information processing among participants with low- or mid-level education.

The main objective of this study was to test the short-term effectiveness of a stand-alone, fully automated, web-based, computer-tailored PA intervention on MVPA in adults. The secondary objectives were: (1) to assess the effectiveness of the intervention according to different PA levels at baseline and sex, (2) to analyze whether the completion of all web sessions varied in relation to participants’ sociodemographic and PA behavioral characteristics, and (3) to determine whether video-delivery tailored PA feedback information processing varied among participants with different education levels.

## Method

### Study design, population, and randomization

The study was a parallel randomized controlled trial (ISRCTN 36353353), with the experimental arm receiving the web-based computer-tailored intervention and the control arm receiving only the same baseline and final evaluations as the experimental group. The control group received the intervention after the trial. Details concerning the design, recruitment and population have been described previously (Boudreau et al., 2015).

The participants were men and women from urban and rural Quebec City who were enrolled in the international Prospective Urban and Rural Epidemiological (PURE) study (Teo, Chow, Vaz, Rangarajan, & Yusuf, 2009). The eligibility criteria for the present study included: (1) age between 35 and 70 years; (2) no history of cardiovascular disease; (3) no limitations concerning the ability to walk; and (4) familiarity with and home access to a computer. At first, the eligibility criteria included only participants who did not reach the recommended 150 min of MVPA weekly. Following the assessment of the baseline recruitment, the research team decided to also include participants achieving the Canadian physical activity guidelines.

In April 2014, potential participants received an email communication describing the study and an electronic participation form. Interested participants were invited to complete and return the form to the center. A follow-up reminder email was sent to non-respondents one week after the invitation. There was no other intervention or promotion for the recruitment of potential participants.

Excel software (RAND function) was used by a member of the research team (A-S B) to randomize eligible participants to the intervention or control arm. Men and women living together were assigned to the same arm based on the first spouse’s randomized allocation. Participants were stratified based on their cardiovascular risk (low, moderate, high) according to the INTERHEART Modifiable Risk Score (McGorrian et al., 2011). Three members of the research team (FB, GT, A-S B) and participants were not blinded to group allocation. The study protocol was approved by the ethics committees of the Université du Québec à Trois-Rivières and the Institut Universitaire de Cardiologie et de Pneumologie de Québec. Each participant received information on the study objective and protocol and gave their informed consent online via the study website during the registration process.

### Web-based computer-tailored intervention

Automatization of the tailoring process was realized with an online tool offering advanced possibilities called TailorBuilder (OverNite Software Europe). Through logical algorithms, the participant received tailored feedback from a set of previously formulated feedback that matched with the online questionnaire scores on PA behavior and determinants regarding behavior (Romeike, Lechner, de Vries, & Oenema, 2016), which was completed during the first session. More specifically, the TailorBuilder is a tool that, by inputting questions, feedback and formulas, generates tailor-made advice. The TailorBuilder is fully web-based. This means that both the development of the intervention by the research team and the intervention itself occurred via the Internet. The advantage of this medium is that the TailorBuilder was available on every computer with an Internet connection. Another advantage is that the TailorBuilder never has to be installed.

The development of the fully automated web-based computer-tailored intervention was based on the I-Change Model (de Vries, Kremers, Smeets, Brug, & Eijmael, 2008) which incorporates several socio-cognitive models, including the theory of planned behavior (Ajzen, 1991), the social-cognitive theory (Bandura, 1986), the transtheoretical model (Prochaska & Velicer, 1997), and the goal-setting theory (Locke & Latham, 1994). The intervention content consisted of seven sessions requiring approximately 10–15 min each to complete, which could be performed over a period of eight weeks (see Table 1 for participants’ progress through the weeks). For each of the sessions, participants were invited to log in with a username and password provided by the research team during the registration process. To remind participants that they could continue with the next session, an automated email was sent seven days after session completion. In the case of a non-response, a reminder email was sent 10 days after session completion. Participants received tailored feedback on their physical activity level and the following I-Change Model determinants: knowledge, risk perception, attitude, social influence, self-efficacy, and PA intention. For instance, for PA intention, there were three possible tailored feedbacks, depending on the expressed level of intent to engage in regular PA: low, moderate and high intent. For those characterized by a low intention, the message was as follows:

Dear [participant name], you have indicated that you do not intend to be regularly physically active in the next month. This is entirely understandable! It can be difficult to be regularly physically active and sometimes it takes a lot of effort. Let’s take a closer look together at why you don’t intend to be regularly physically active.

Subsequently, on the same principle of low, moderate, and high scores, the participant received tailored feedback related to their attitude, social influence, and self-efficacy. Furthermore, women received feedback on additional beliefs particularly relevant to them (e.g. not having sufficient time to exercise because of family responsibilities). Likewise, during the intervention, instead of text, participants with low-level (less than a high school diploma) to mid-level (high school diploma) education received tailored feedback regarding their physical activity level and their I-Change Model beliefs via video delivery. A total of 33 video-delivery tailored feedback recordings were created by Point Bleu Productions (Trois-Rivières, Canada). Table 1 presents an overview of the content of each session. However, a previous publication presents in more detail the content of each of the web sessions (Boudreau et al., 2015).

**Table 1.** Overview of the web-based computer-tailored physical activity intervention sessions.

**Session 1 (week 1)**
• Explanation of the study
• Obtaining informed consent for taking part in the study
• Baseline measurement: demographics, physical activity level, and I-Change Model variables
**Session 2** (immediately after session 1 or wait a few days before starting the session).
• Introduction of the session: explanation of the intervention and the different sessions
• Tailored feedback related to participant's belief of the benefits of physical activity on their heart
• Optional: information about the Canadian guidelines and the different intensity categories of physical activity
• Tailored feedback on physical activity level^a^
• Tailored feedback on attitude^a,b^
• Summary of session
**Session 3** (one week after session two)
• Introduction of the session
• Tailored feedback on intention^a^
• Tailored feedback on self-efficacy
• Tailored feedback on social influence (social support, modelling, and social norm)^a^
• Optional: tailored feedback on attitude (same as in session 1)^a,b^
• Goal setting: selection of amount of physical activity increase (10–60 min per week)
• Change date: setting date when starting with goal (7–14 days after session 3)
**Session 4** (four days before the day they want to increase their moderate-to vigorous-intensity PA level)
• Introduction of the session
• Information about the use of preparatory plans
• Assessment of self-efficacy towards achieving goal
• Tailored feedback on self-efficacy towards achieving goal
• Assessment of self-efficacy towards achieving goal in difficult situations
• Tailored feedback on self-efficacy towards achieving goal in difficult situations
• Summary of the session
**Session 5** (one week after the change date)
• Introduction of the session
• Assessment physical activity level during past week
• Tailored feedback on physical activity progress^a^
• Optional: tailored feedback on intention, self-efficacy, social influence, and attitude (identical to session 2)
• Coping planning: assessment of difficult situations^b^
• Coping planning: tailored feedback on selected difficult situation(s)^b^
• Coping planning: selection of coping option(s)^b^
• Summary of the session
**Session 6** (two weeks after session 5)
• Identical to session 5
• Additional: information about how to main behavior changes on long-term
• Summary of the session
**Session 7** (four weeks after session 5)
• Identical to session 6
• Additional: process evaluation

Note: ^a^This part was delivered using videos for people with a low educational level; ^b^This part includes additional information for women.

### Measures

#### Participation in moderate-to-vigorous physical activity

Participants in both arms of the study underwent a standardized evaluation at baseline and at a 3-month follow-up. A slightly modified version of the validated Godin-Shephard Leisure Time Physical Activity Questionnaire was used (Godin & Shephard, 1985). Participants were asked to self-report: (1) the frequency of their practiced intensity-specific PA (i.e. light, moderate, vigorous) in a typical seven-day period and (2) the average duration, in minutes, of these activities for each intensity category. In the original questionnaire, the duration of PA sessions was expressed as ‘more than 15 minutes.’ Greater precision concerning the duration of intensity-specific PA should strengthen the assessment of the behavior. The procedure for calculating the total minutes of MVPA per week was as follows. First, the weekly duration in minutes of each category of intensity-specific physical activities was calculated separately by multiplying the frequency of days per week by the activity duration. The reported minutes per week in each category were then weighted by a metabolic equivalent (MET) energy expenditure estimate. Light-, moderate-, and vigorous-intensity activities were considered equivalent to 3.3, 4, and 8 METs, respectively (IPAQ, 2005). Finally, the weighted MET-minutes per week (MET-min/week) were calculated for each category of intensity-specific physical activities as follows: (duration/session) × (frequency of sessions/week) × (MET intensity), where a score greater than or equal to 600 MET-min/week was equivalent to 150 min of MVPA per week (Detry & Ma, 2016).

#### Psychosocial determinants

Participants answered questions regarding some of the I-Change Model variables on the study website. Table 2 (panel A) describes each of these variables and their psychometric values. All variables were assessed at the baseline; however, only intention and self-efficacy were reassessed after three months to avoid a lengthy follow-up questionnaire. This choice was based on a Canadian study that showed support for a significant relationship for each of these two determinants with changes in PA (Plotnikoff, Lubans, Trinh, & Craig, 2012).

**Table 2.** Description of the psychosocial (panel A) and processing of information variables (panel B).

Variables	Seven-point scales	Internal consistency^a^
**Panel A – Psychosocial variables**		
*Intention*		0.76^b^
• I intend to practice regularly one or more physical activities in my free times within the next month	Unlikely/likely	
• My plans are to practice regularly one or more physical activities in my free times within the next month	Disagree/agree	
*Attitude-pros of physical activity* (example below for one the seven items)		0.79
• If I practiced regularly one or more physical activities in my free times within the next month, I would have a better control of my weight	Unlikely/likely	
*Attitude-cons of physical activity* (example below for one of the six items)		0.69
• If I practiced regularly one or more physical activities in my free times within the next month, I would miss time to do other things	Unlikely/likely	
*Social influence* (example below for one of the four items)		0.84
• My physician/wife/husband encourage/support me to do regular physical activity	Disagree/agree	
*Social norm*		0.87^b^
• According to people who are part of your daily life, you should be physically active on a regular basis	Disagree/agree	
• People who are part of your daily life think that it is important for you to be physically active on a regular basis	Disagree/agree	
*Modeling*		0.66^b^
• Most people who are part of your daily life are physically active on a regular basis	Disagree/agree	
• Among the people who are part of your daily life, how many are physically active on a regular basis?	None/everybody	
*Self-efficacy* (example below for one of the thirteen items)		0.91
**•** I am able to be sufficiently physically active when the weather is bad		
**Panel B – Processing of information variables**		
*Attention for the tailored feedback*		0.90
• The feedback and information attracted my attention	Disagree/agree	
• The feedback and information kept my attention	Disagree/agree	
• The feedback and information were attractive	Disagree/agree	
*Comprehension of the tailored feedback*		0.80
• The feedback and information were clear.	Disagree/agree	
• The feedback and information were understandable.	Disagree/agree	
• The feedback and information were hard to understand.	Disagree/agree	
*Appreciation of the tailored feedback*		0.95
• The feedback and information were interesting	Disagree/agree	
• The feedback and information were useful	Disagree/agree	
• The feedback and information were motivating	Disagree/agree	
• I liked the feedback and information	Disagree/agree	
• The feedback and information suited me well		
*Persuasion of the tailored feedback*		0.84
• The feedback and information were convincing	Disagree/agree	
• The feedback and information were credible	Disagree/agree	
• The feedback and information were reliable	Disagree/agree	

Note: ^a^Internal consistency was reported as Cronbach’s alpha coefficient for variables of 3 items or more; ^b^Spearman’s correlation coefficient for variables of 2 items.

#### Processing of information

Participants were invited to complete the assessment during the seventh and last web session. Those who did not were invited to do so during the 3-month follow-up. The information was assessed using four variables, as described in Table 2 (panel B). In addition, all participants were asked to provide an overall evaluation of the intervention on a 10-point scale ranging from ‘very bad’ (+1) to ‘very good’ (+10). Assessment regarding the video-delivery tailored feedback was also evaluated for participants with low- or mid-level education.

### Sample size

Sample size was calculated using the G*Power software platform (Faul, Erdfelder, Lang, & Buchner, 2007). Assuming a power of 0.80, an alpha value of 0.05 and a moderate correlation (*r* = 0.5) between baseline and 3-month follow-up PA measures, it was determined that a total sample size of 200 participants was required to detect a small effect size (*f* = .10) based on Cohen’s convention (Cohen, 1992). This effect size was chosen in accordance with findings reported in the field of computer-tailored interventions (Krebs et al., 2010). Participants invited to the present study (*n* = 1269) should provide flexibility to obtain the sufficient statistical power at the 3-month follow-up, based on an expected attrition rate of 40% (Peels et al., 2012).

### Statistical analysis

The 3-month effectiveness of the intervention for increasing weekly minutes of self-reported MVPA was assessed with repeated measures ANOVA using a linear mixed model. Linear mixed models can properly account for correlations among repeated measurements on the same subject and have greater flexibility to model time effects (Gueorguieva & Krystal, 2004). Moreover, linear mixed models are better able to handle missing observations (Peters et al., 2012; Twisk, de Boer, de Vente, & Heymans, 2013). Multiple imputation was, however, carried out as a sensitivity analysis in order to verify the robustness of the findings based on the linear mixed model analysis (Thabane et al., 2013).

The model considered a within-subject fixed effect (baseline, 3-month follow-up), a between-subject fixed effect (intervention group, control group), a two-way interaction effect ‘time-by-group’ and the addition of a random effect (subject effect nested within the group effect). Furthermore, potential interaction effects due to ‘sex-by-group,’ ‘baseline PA level-by-group,’ ‘education level-by-group,’ and ‘age-by-group’ were added to the model to ensure that the effectiveness of the intervention on self-reported MVPA was not dependent on participants’ baseline PA behavior or socio-demographic characteristics. The covariance structure was tested, and the heterogeneous compound symmetry covariance matrix appeared to be the most adequate for the model based on Akaike’s Information Criterion (Bozdogan, 1987). Minutes of MVPA were positively skewed and were square root-transformed for the analysis. The residual plot was visually inspected and was not observed to obviously deviate from homoscedasticity or normality. For a better understanding of the results, the non-transformed mean scores have been used. The analysis was adjusted for baseline covariates (Deaton & Cartwright, 2018). The same analysis was repeated, but this time by evaluating the 3-month effectiveness of the intervention on two key psychosocial determinants susceptible to influence the primary outcome, namely ‘intention to practice regular MVPA’ and ‘sense of self-efficacy over regular MVPA’. These two determinants are borrowed from the theoretical framework, the I-Change model, and were targeted by the intervention. To verify whether the completion of all web sessions differed according to participants’ characteristics, a binary logistic regression was conducted with intervention completion (0 = no/1 = yes) as the dependent variable and sex, age, level of education, PA intention at baseline, and achieving (or not) PA guidelines as the predictors of intervention completion. Likewise, to verify whether the number of web sessions completed had an effect on self-reported MVPA, a post-hoc trend analysis was performed by means of a linear polynomial contrast using an ANOVA. To identify any difference between participants with low- or mid-level education and high-level education and the impact of exposure to video-delivery and text-delivery tailored feedback, respectively, on attention for tailored feedback, comprehension, appreciation, and persuasion, four 1-way ANOVAs with a Bonferroni correction were performed.

The significance levels were set at *p* < .05 for all analyses, except for testing the interaction terms (*p* < .10), which have less power (Twisk, 2006). The software Statistical Analysis System (SAS version 9.4, SAS Institute) was used to perform the statistical analyses in line with the study’s objectives.

## Results

### Study population

**Figure 1.**
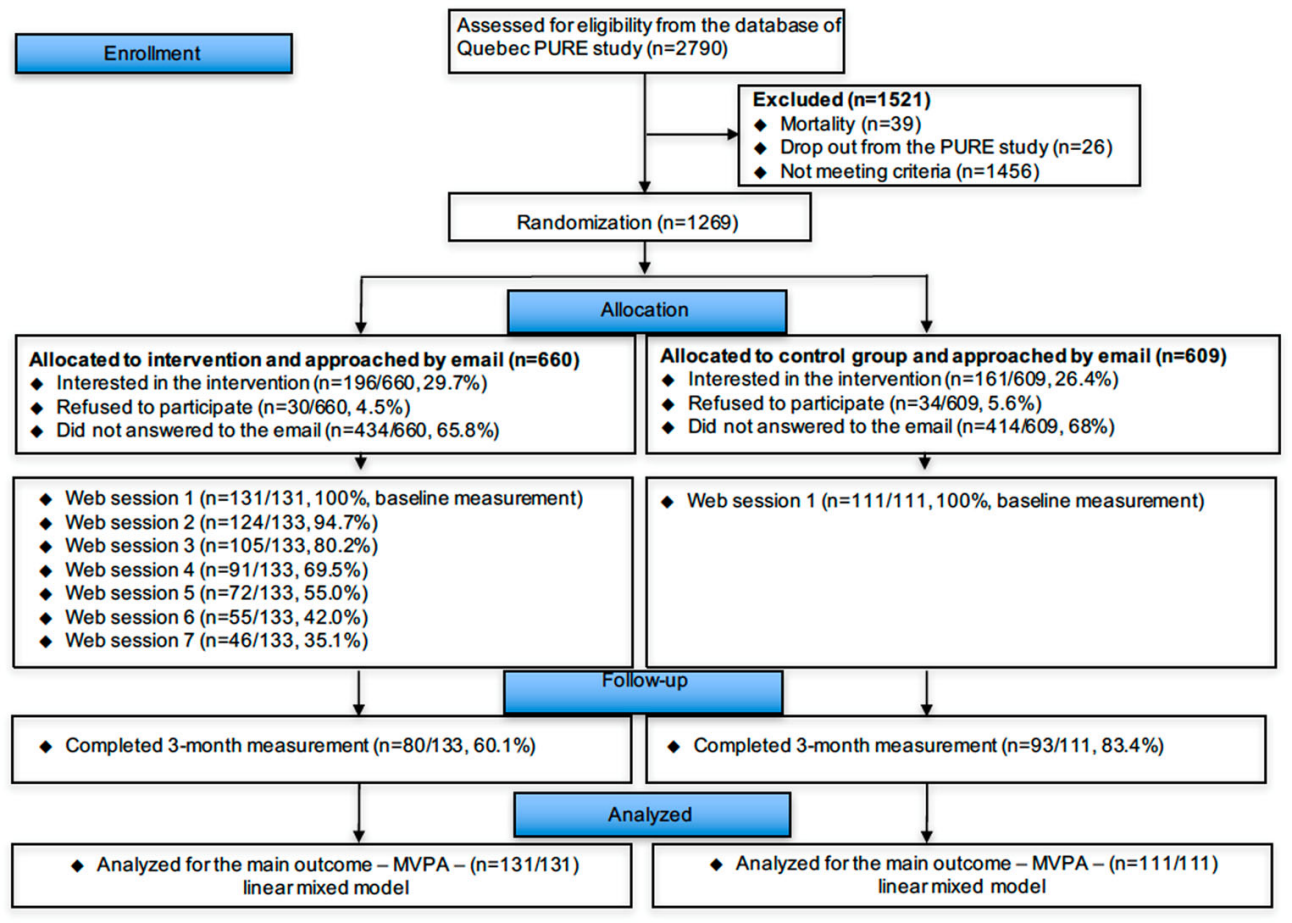
CONSORT flow chart of participants through the study.

Of the 2,790 participants, 1,269 met the eligibility criteria and were randomized to the intervention (*n* = 660) or control (*n* = 609) arm. Of the 660 participants randomized to the intervention group, 434 (65.8%) did not reply to the two email invitations, 30 (4.5%) refused to participate, 196 (29.7%) expressed interest, and 131 gave consent and underwent baseline measurements. Of the 609 participants randomized to the control arm, 414 (68.0%) ignored the two email invitations, 34 (5.6%) refused to participate, 161 expressed interest, and 111 gave consent and underwent baseline measurements. Finally, 80 (60%) of the 131 participants in the intervention group and 93 (84%) of the 111 participants in the control group completed the 3-month follow-up (Figure 1).

There were no differences in age, sex, education level, cardiovascular risk factors, INTERHEART modifiable risk score, physical activity intention, or MVPA between the participants in the intervention and those in the control groups (Table 3). In terms of dropouts, participants in the intervention group had 3.5 times higher odds of dropping out of the study than those in the control group (95% CI [1.88–6.67], *p* < .001). Likewise, for each one-year reduction in age, the odds of dropping out of the study increased by a factor of 1.04 (95% CI [1.00–1.08], *p* = .049).

**Table 3.** Characteristics of the randomized participants at baseline.

	All participants (*n* = 242)		
	Control (*n* = 111)	Intervention (*n* = 131)	*p* value
Sociodemographics			
Mean age (*SD*), years	55.6 (7.3)	57.0 (7.3)	.13
Sex, *n* (% within group)			
Women	80 (72.1)	83 (63.4)	.15
Men	31 (27.9)	48 (36.6)	
Highest education level completed, *n* (% within group)			.13
High school (mid-level education)	14 (12.6)	26 (19.8)	
College/university (high-level education)	97 (87.4)	105 (80.2)	
			
Cardiovascular risk factors			
Smoking behaviour, *n* (% within group)			
Current	9 (8.1)	5 (3.8)	
Never	58 (52.3)	68 (51.9)	
Former	44 (39.6)	58 (44.3)	
Mean body mass index (*SD*), kg/m^2^	25.8 (4.8)	26.4 (4.3)	.33
Mean blood pressure (*SD*), mm Hg			
Systolic	126 (18.9)	127.9 (17.8)	.43
Diastolic	78.8 (11.2)	80.2 (11)	.33
Mean low-density lipoprotein level (*SD*), mmol/L	2.9 (.74)	3.1 (.85)	.29
Mean high-density lipoprotein level (*SD*), mmol/L	1.7 (.43)	1.7 (.46)	.90
Mean apolipoprotein B level (*SD*), mmol/L	.92 (.23)	.91 (.24)	.66
Mean triglycerides level (*SD*), mmol/L	1.2 (.73)	1.2 (.70)	.99
Mean glycemia level (*SD*), mmol/L	5 (.61)	5 (.83)	.88
Cardiovascular modifiable risk score^a^*n* (% within group)			
Low	35 (31.5)	41 (31.3)	
Moderate	48 (43.2)	57 (43.5)	
High	28 (25.2)	33 (25.2)	
Physical activity			
Mean physical activity intention (*SD*), score varying from +1 (very low) to +7 (very high).	5.9 (1.6)	6.1 (1.4)	.25
Mean sense of self-efficacy over physical activity (*SD*), score varying from +1 (disagree) to +7 (agree).	5.1 (1.1)	5.2 (1.1)	.45
Mean moderate-to-vigorous physical activity behaviour (*SD*), minutes per week	147.6 (174.0)	158.3 (213.8)	.67

Note: ^a^According to the INTERHEART modifiable risk score (McGorrian et al., 2011).

### Intervention effects on moderate-to-vigorous physical activity and psychosocial determinants

**Figure 2.**
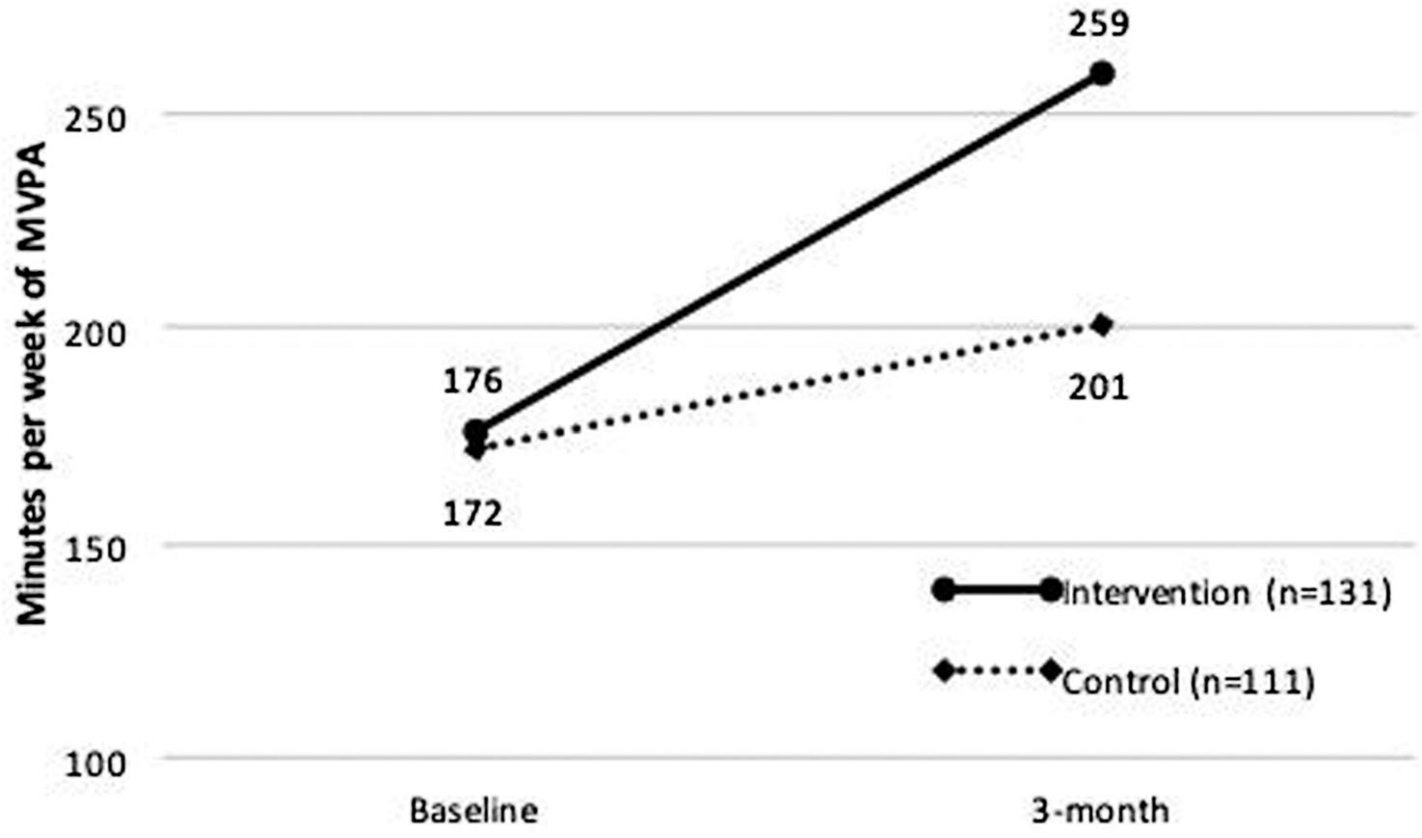
Estimated marginal means of weekly minutes of moderate-to-vigorous physical activity for intervention and control groups at baseline and at 3-month follow-up. *Significant difference between the intervention and control groups. MVPA, moderate-to-vigorous physical activity.

A linear mixed model-repeated measures ANOVA showed a significant ‘group-by-time’ interaction, *F*(1, 167) = 5.37, *p *= .02, indicating that participants in the intervention group experienced a significantly larger increase in self-reported weekly minutes of MVPA than participants in the control group. The sensitivity analysis using multiple imputation produced comparable results, *t*(1596.3) = 1.76, *p*_interaction_ = .08. Figure 2 shows the differences between the study groups at each time point. At baseline, the self-reported weekly minutes of MVPA did not differ significantly between the intervention and the control group (mean ± SD; 176 ± 13 vs. 172 ± 15 min/week), *F*(1, 181) = 0.14, *p *= .72. In contrast, at the final 3-month follow-up, weekly minutes of MVPA were significantly higher in the intervention group than in the control group (259 ± 21 vs. 201 ± 22 min/week), *F*(1, 175) = 4.04, *p *= .04. There were no significant interaction effects on average weekly minutes of MVPA for ‘sex-by-group’ (*p* = .57), ‘baseline PA level-by-group’ (*p *= .43), ‘education level-by-group’ (*p* = .92), or ‘age-by-group’ (*p* = .69). All main effects on average weekly minutes of MVPA were not statistically significant (education, *p* = .74; sex, *p* = .95; age, *p* = .21; group, *p* = .87), except for the main effect of participants’ baseline PA (*p* < .001).

As for the two key psychosocial determinants, no significant ‘group-by-time’ interaction was found for ‘intention to practice regular MVPA’, *F*(1, 218) = 0.47, *p *= .50, and ‘sense of self-efficacy over regular MVPA’, *F*(1, 205) = 0.26, *p *= .61.

### Participation levels within the intervention group

Participation in the different sessions varied, as follows: session 1 (*n* = 131, 100%), session 2 (*n* = 124, 95%), session 3 (*n* = 105, 80%), session 4 (*n* = 91, 70%), session 5 (*n* = 72, 55%), session 6 (*n* = 55, 42%), and session 7 (*n* = 46, 35%). Of the five predictors of intervention completion, defined as participating in all seven web sessions, four were statistically significant: participants meeting PA recommendations at baseline, OR = 3.32, 95% CI [1.44–7.66], *p* = .005; participants having a mid-level education, OR = 2.78, 95% CI [1.04–7.42], *p* = .04; participants being women, OR = 2.34, 95% CI [1.00–5.46], *p* = .049; and participants being older, with a one-year increase in age increasing the odds of participating in all seven web sessions by a factor of 1.08, 95% CI [1.02–1.14], *p* = .01.

To analyze whether the number of web sessions completed has an effect on self-reported MVPA, a post-hoc trend analysis was performed by means of a linear polynomial contrast using an ANOVA. Participants completing more web sessions had a higher MVPA, *F*(1, 77) = 4.74, *p* < .05. The difference between baseline and 3-month assessments was an increase of 3.9 min per week for participants who completed sessions one through three and increases of 46.8 and 78.9 min per week for those who completed sessions four through five and six through seven, respectively.

### Processing of information for video-tailored feedback and text-tailored feedback

As shown in Table 4, the information processing indicates that participants in the intervention group scored relatively high on attention, comprehension, appreciation, and persuasion of the tailored feedback. No significant differences were observed between participants exposed to the video-tailored feedback (mid-level education) and those exposed to the text-tailored feedback (high-level education) regarding the aforementioned concepts (all *p*s > .05). With respect to the overall evaluation of the intervention, no significant difference was observed between participants exposed to the video-tailored feedback (*M *= 8.05, *SD *= ±1.50) and those exposed to the text-tailored feedback (*M *= 7.61, *SD *= ±1.69), *F*(1,89) = 1.10, *p* = .30. Finally, participants with mid-level education expressed that the pace of the video-tailored feedback was ‘near perfect,’ ‘agreed’ that it was pleasant to listen to the people who gave the advice in the videos, and found the experience of receiving feedback and information via videos ‘pleasant’ (Table 4).

**Table 4.** Processing of information according to the type of tailored delivery feedback.

	All Participants		Participants receiving video-tailored feedback (*n *= 20)		Participants receiving text-tailored feedback (*n *= 71)		*F*	*p*
	Mean	SD	Mean	SD	Mean	SD		
Attention for the tailored feedback^a^	5.66	1.23	5.66	1.48	5.66	1.16	0.00	.99
Comprehension of the tailored feedback^a^	4.98	0.51	5.13	0.71	4.94	0.43	2.34	.13
Appreciation of the tailored feedback^a^	5.75	1.16	6.04	0.97	5.68	1.20	1.55	.22
Persuasion of the tailored feedback^a^	5.91	1.03	6.18	0.83	5.83	1.07	1.85	.18
Overall assessment of the intervention^b^	7.71	1.65	8.05	1.50	7.61	1.69	1.10	.30
How did you experience the pace at which the feedback and information were given in the videos?^c^	–	–	3.94	0.66	–		–	–
The people who gave the advices in the videos were pleasant to listen to/watch^a^	–	–	6.29	0.77	–		–	–
How did you experience receiving feedback and information via videos?^d^	–	–	6.29	1.21	–		–	–

Note: ^a^Scores varied between +1 (strongly disagree), +7 (strongly agree); ^b^Scores varied between +1 (very bad), +10 (very good); ^c^Scores varied between +1 (much too fast), +4 (perfect pace), +7 (much too slow); ^d^Scores varied between +1 (totally not pleasant) to +7 (very pleasant).

## Discussion

This randomized controlled trial was mainly conducted to determine the effectiveness of a web-based computer-tailored PA intervention at a 3-month follow-up. Although recruitment and engagement were lower than expected, the intervention was associated with a significant increase in self-reported MVPA relative to the control group. Predictors regarding participation in all seven web-based sessions were: meeting PA recommendations at baseline, having a mid-level education, being a woman, and being older. In addition, the more web sessions participants achieve, the greater their self-reported MVPA tended to be at the 3-month follow-up. Finally, regardless of the type of delivery feedback (video- or text-delivery), participants scored relatively high on different dimensions of information processing.

In randomized studies using computer-tailoring technology targeting healthy adults, Plaete et al. (2015) also found an increase in moderate intensity PA at a 1-month follow-up, while Compernolle, Vandelanotte, Cardon, De Bourdeaudhuij, and De Cocker (2015) found no significant increase in MVPA at a 3-month follow-up. While these studies had objectives other than testing whether the effectiveness of the interventions was dependent on participants’ socio-demographic characteristics, the present study adds new knowledge in the field of web-based computer-tailored PA interventions. Indeed, the findings suggest that the used intervention could be implemented to promote regular MVPA in women and men of all ages with both mid- and high-level education, meeting or not meeting physical activity guidelines. These results are in line with those of Cheung et al. (2017), who highlighted that the effectiveness of a video-delivery tailored intervention on average daily energy intake after one year was not dependent on participants’ educational levels or body mass index.

For the two key psychosocial determinants that may influence MVPA participation, namely intention and self-efficacy, no significant difference was observed between participants in the intervention and control groups at 3-month follow-up. As a possible explanation, for ‘intention to practice regular MVPA’, the mean baseline score was quite high in both groups; therefore, due to this ceiling effect, a more pronounced increase for participants in the experimental group compared with the control group was rather difficult. An alternative explanation is that other determinants of the I-Change model, not assessed at 3-month follow-up, such as attitude and social influence, could explain the difference in self-reported MVPA participation between the two groups of participants. Finally, as meta-analyses have already suggested, intention alone is insufficient to enable participants to act on their behavior (Rhodes & Dickau, 2012; Webb & Sheeran, 2006). In the context of the present study, to bridge the intention-behavior gap for the participants in the experimental group, behavioral self-regulation techniques were used during Web-based sessions 3 (e.g. preparatory planning, goal setting) and 4 (e.g. tailored feedback on PA progress, coping planning). The use of these techniques could explain the difference in self-reported MVPA participation between the two groups of participants at 3-month follow-up. However, the study was not designed to assess the effectiveness of these self-regulation techniques.

Although the pattern of change in self-reported MVPA was independent of participants’ behaviors at baseline, participants who did not meet PA recommendations at baseline significantly increased minutes of MVPA following exposure to the web-based tailored intervention. A recent meta-analysis also highlighted the short-term effectiveness of PA interventions among participants who did not meet PA recommendations at baseline (Howlett, Trivedi, Troop, & Chater, 2019); however, most of the studies selected involved direct contacts with the participants (face-to-face, telephone call, supervised training, etc.). Relative to those conventional interventions, one of the key advantages of the present web-based tailored intervention is its personalized yet automated nature. Like Arem et al.’s meta-analysis (Arem et al., 2015), the present study points out the importance of delivering messages designed specifically for participants who do not meet PA guidelines at baseline; these participants could benefit the most from increasing their behavior to reduce their risk of cardiovascular disease.

Like participants who did not meet PA guidelines at baseline, women exposed to the web-based tailored intervention significantly increased their level of self-reported MVPA. As there is insufficient evidence to assess the effectiveness of community-based interventions to enhance PA among women (Amiri Farahani et al., 2015), the current findings suggest the relevance of an intervention designed specifically for women to efficiently promote regular MVPA. While other internet-based PA interventions in middle-aged women have also been effective (Lieber et al., 2012; Peyman et al., 2018), promoting regular PA specifically for this group through eHealth interventions continued to pose a challenge (Reed et al., 2015). In this way, the present study contributes to our knowledge regarding the potential of computer-tailoring technology to promote PA among women.

In accordance with other studies, the present study experienced documented declining engagement to the seven web-based sessions (Edney et al., 2019; Friederichs, Oenema, Bolman, & Lechner, 2016; Reinwand et al., 2016; Storm et al., 2016; Voncken-Brewster et al., 2015; Walthouwer, Oenema, Lechner, & de Vries, 2015). This non-engagement is not unique to web-based computer-tailored PA interventions; online social network interventions also suffer low participant commitment (Maher et al., 2014). In fact, commitment toward (or re-use of) the current web-based computer-tailored intervention is consistent with the idea that one of the most substantial issues in e-health technologies is participants’ engagement (Barello et al., 2016; Kohl, Crutzen, & de Vries, 2013; Perski, Blandford, West, & Michie, 2017; Yardley et al., 2016). During the present intervention, personalized and automated emails inviting non-adherent participants to complete the web sessions did not suffice to maintain participant engagement throughout the intervention, despite evidence that this effectively increases engagement (Schneider, de Vries, Candel, van de Kar, & van Osch, 2013). However, regardless of the strategies used, one of the critical aspects of participants’ engagement in an eHealth intervention aimed at the adoption of healthy lifestyles is to fully appreciate participants’ needs and perspectives by involving them in the development of the intervention (Yardley et al., 2016). Within the context of the present study, the intervention had to be developed, implemented and evaluated within one year due to the short funding period. This made it difficult to optimally implement methods to better understand the needs and perspectives of participants (e.g. focus groups, usability testing), which might explain in part the observed disengagement. Many experts believe that, ultimately, the issue of participants’ engagement can be explained by the fact that their needs and perspectives are not taken into account when designing an eHealth intervention (Birnbaum, Lewis, Rosen, & Ranney, 2015). Considering our data suggesting that participating in all web sessions improves the effect on practice of self-reported MVPA, future studies should use innovative ideas to foster participants’ engagement. More precisely, based on the conceptual framework of Cole-Lewis, Ezeanochie, and Turgiss (2019), innovative ideas for fostering engagement could be directed towards (1) improving interactions with intervention features designed to encourage frequency of use and make the participant experience attractive and (2) improving participant interactions with the components of behavior change intervention that influence the determinants of health behavior and subsequently influence health behavior.

No difference was observed in information processing between participants with mid-level education exposed to video-delivery tailored feedback and those with high-level education exposed to text-delivery tailored feedback. These data must be interpreted cautiously, as the participants with mid-level education exposed to video-tailored feedback should have been compared with other participants with mid-level education exposed to text-video feedback. However, due to the small number of participants with m16id-level education, the comparison was performed with participants with high-level education.

## Study limitations and strengths

Only the short-term effect was assessed in this study due to the duration of the research grant (i.e. one year). Second, while the sample size needed for the 3-month follow-up was estimated at 200 participants, 183 participants actually completed the follow-up questionnaire. Therefore, the participation rate for the 3-month follow-up was less than estimated. It is possible that a more optimal recruitment and retention strategies would have resulted in a larger sample size at 3 month follow up (Watson, Mull, Heffner, McClure, & Bricker, 2018). Other limitations include the study’s reliance on self-reporting using the Godin-Shephard Leisure Time Physical Activity Questionnaire. The reliability and validity of the original questionnaire compared favorably to nine other self-report measurements (Jacobs, Ainsworth, Hartman, & Leon, 1993); however, it is unknown whether the current study’s slight change to the questionnaire affects its measurement qualities, given the absence of studies providing validity evidence supporting the use of this modified form (Amireault & Godin, 2015). Likewise, the use of a subjective method (i.e. self-report) to assess PA is a limitation. Ideally, an objective method would have been appropriate to complement the subjective one. Both of these methods mitigate their respective limitations in measuring PA (Garriguet & Colley, 2014). Finally, the intervention was only available online, thus excluding participants without access to the internet, who are more likely to have low-level education. The generalization of our findings is, thus, limited.

Nevertheless, our study has strengths. First, the primary analysis regarding the effectiveness of the intervention was corroborated by the sensitivity analysis. The difference in *p*-value between the primary analysis (linear mixed model) and sensitivity analysis (multiple imputation) methods could be explained in part by the fact that the former offers greater statistical power (Sullivan, White, Salter, Ryan, & Lee, 2018; Xi, Pennell, Andridge, & Paskett, 2018). Second, the use of video-delivery tailored feedback in the context of web-based computer-tailored interventions is innovative.

## Conclusion

The findings of our study suggest that the used PA intervention is effective in enhancing self-reported MVPA in an adult population in the short term. Innovative approaches and better active promotion must be developed to recruit more potential participants to visit the website, to register, and to enhance engagement to all sessions. Finally, while video-delivery tailored feedback seems to be a promising avenue to facilitate information processing in individuals with mid-level education, further research is needed to confirm our results concerning information processing and delivering tailored, customized feedback.

## References

[CIT0001] Ajzen, I. (1991). The theory of planned behavior. *Organizational Behavior and Human Decision Processes*, *50*, 179–211.

[CIT0002] Alley, S., Jennings, C., Persaud, N., Plotnikoff, R. C., Horsley, M., & Vandelanotte, C. (2014). Do personally tailored videos in a web-based physical activity intervention lead to higher attention and recall? – An eye-tracking study. *Frontiers in Public Health*, *2*, 13. doi:10.3389/fpubh.2014.00013 24575398 PMC3921670

[CIT0003] Amireault, S., & Godin, G. (2015). The Godin-Shephard leisure-time physical activity questionnaire: Validity evidence supporting its use for classifying healthy adults into active and insufficiently active categories. *Perceptual and Motor Skills*, *120*(2), 604–622. doi:10.2466/03.27.PMS.120v19x7 25799030

[CIT0004] Amiri Farahani, L., Asadi-Lari, M., Mohammadi, E., Parvizy, S., Haghdoost, A. A., & Taghizadeh, Z. (2015). Community-based physical activity interventions among women: A systematic review. *BMJ Open*, *5*(4), e007210–e007210. doi:10.1136/bmjopen-2014-007210 PMC4390687 25833668

[CIT0005] Arem, H., Moore, S. C., Patel, A., Hartge, P., Berrington de Gonzalez, A., Visvanathan, K., … Matthews, C. E. (2015). Leisure time physical activity and mortality: A detailed pooled analysis of the dose-response relationship. *JAMA Internal Medicine*, *175*(6), 959–967. doi:10.1001/jamainternmed.2015.0533 25844730 PMC4451435

[CIT0006] Bandura, A. (1986). *Social foundations of thought and action: A social cognitive theory*. Englewood Cliffs, NJ: Prentice-Hall.

[CIT0007] Barello, S., Triberti, S., Graffigna, G., Libreri, C., Serino, S., Hibbard, J., & Riva, G. (2016). Ehealth for patient engagement: A systematic review. *Frontiers in Psychology*, *6*, 2013. doi:10.3389/fpsyg.2015.02013 26779108 PMC4705444

[CIT0008] Bennett, G. G., & Glasgow, R. E. (2009). The delivery of public health interventions via the Internet: Actualizing their potential. *Annual Review of Public Health*, *30*, 273–292. 10.1146/annurev.publhealth.031308.100235 19296777

[CIT0009] Birnbaum, F., Lewis, D., Rosen, R. K., & Ranney, M. L. (2015). Patient engagement and the design of digital health. *Academic Emergency Medicine*, *22*(6), 754–756. doi:10.1111/acem.12692 25997375 PMC4674428

[CIT0010] Boogerd, E. A., Arts, T., Engelen, L. J., & van de Belt, T. H. (2015). “What is eHealth”: Time for an update? *JMIR Research Protocols*, *4*(1), e29. doi:10.2196/resprot.4065 25768939 PMC4376129

[CIT0011] Boudreau, F., Walthouwer, M. J. L., de Vries, H., Dagenais, G. R., Turbide, G., Bourlaud, A.-S., … Poirier, P. (2015). Rationale, design and baseline characteristics of a randomized controlled trial of a web-based computer-tailored physical activity intervention for adults from Quebec City. *BMC Public Health*, *15*(1), 1038. doi:10.1186/s12889-015-2364-3 26453041 PMC4600320

[CIT0012] Bozdogan, H. (1987). Model selection and Akaike’s information criterion (AIC): The general theory and its analytical extensions. *Psychometrika*, *52*(3), 345–370. doi:10.1007/BF02294361

[CIT0013] Broekhuizen, K., Kroeze, W., van Poppel, M. N. M., Oenema, A., & Brug, J. (2012). A systematic review of randomized controlled trials on the effectiveness of computer-tailored physical activity and dietary behavior promotion programs: An update. *Annals of Behavioral Medicine*, *44*(2), 259–286. doi:10.1007/s12160-012-9384-3 22767052 PMC3442159

[CIT0014] Cheung, K. L., Schwabe, I., Walthouwer, M. J. L., Oenema, A., Lechner, L., & de Vries, H. (2017). Effectiveness of a video-versus text-based computer-tailored intervention for obesity prevention after one year: A randomized controlled trial. *International Journal of Environmental Research and Public Health*, *14*(10), 1275. doi:10.3390/ijerph14101275 PMC5664776 29065545

[CIT0015] Chua, H. F., Ho, S. S., Jasinska, A. J., Polk, T. A., Welsh, R. C., Liberzon, I., & Strecher, V. J. (2011). Self-related neural response to tailored smoking-cessation messages predicts quitting. *Nature Neuroscience*, *14*(4), 426–427. doi:10.1038/nn.2761 21358641 PMC3109081

[CIT0016] Chua, H. F., Polk, T., Welsh, R., Liberzon, I., & Strecher, V. (2009). Neural responses to elements of a web-based smoking cessation program. *Studies in Health Technology and Informatics*, *144*, 174–178. 19592758 PMC2892852

[CIT0017] Cohen, J. (1992). A power primer. *Psychological Bulletin*, *112*(1), 155–159. doi:10.1037/0033-2909.112.1.155 19565683

[CIT0018] Cole-Lewis, H., Ezeanochie, N., & Turgiss, J. (2019). Understanding health behavior technology engagement: Pathway to measuring digital behavior change interventions. *JMIR Formative Research*, *3*(4), e14052. doi:10.2196/14052 31603427 PMC6813486

[CIT0019] Colley, R. C., Garriguet, D., Janssen, I., Craig, C. L., Clarke, J., & Tremblay, M. S. (2011). Physical activity of Canadian adults: Accelerometer results from the 2007 to 2009 Canadian health measures survey. *Health Reports / Statistics Canada, Canadian Centre For Health Information*, *22*(1), 7–14. 21510585

[CIT0020] Compernolle, S., Vandelanotte, C., Cardon, G., De Bourdeaudhuij, I., & De Cocker, K. (2015). Effectiveness of a web-based, computer-tailored, pedometer-based physical activity intervention for adults: A cluster randomized controlled trial. *Journal of Medical Internet Research*, *17*(2), e38. doi:10.2196/jmir.3402 25665498 PMC4342625

[CIT0021] Cotie, L. M., Prince, S. A., Elliott, C. G., Ziss, M. C., McDonnell, L. A., Mullen, K. A., … Reed, J. L. (2018). The effectiveness of eHealth interventions on physical activity and measures of obesity among working-age women: A systematic review and meta-analysis. *Obesity Reviews*, *19*(10), 1340–1358. doi:10.1111/obr.12700 30156044

[CIT0022] Davies, C. A., Spence, J. C., Vandelanotte, C., Caperchione, C. M., & Mummery, W. K. (2012). Meta-analysis of internet-delivered interventions to increase physical activity levels. *International Journal of Behavioral Nutrition and Physical Activity*, *9*(1), 52. doi:10.1186/1479-5868-9-52 PMC3464872 22546283

[CIT0023] Deaton, A., & Cartwright, N. (2018). Understanding and misunderstanding randomized controlled trials. *Social Science & Medicine*, *210*, 2–21. doi:10.1016/j.socscimed.2017.12.005 29331519 PMC6019115

[CIT0024] Detry, M. A., & Ma, Y. (2016). Analyzing repeated measurements using mixed models. *JAMA*, *315*(4), 407–408. doi:10.1001/jama.2015.19394 26813213

[CIT0025] de Vries, H., & Brug, J. (1999). Computer-tailored interventions motivating people to adopt health promoting behaviours: Introduction to a new approach. *Patient Education and Counseling*, *36*(2), 99–105. 10223015 10.1016/s0738-3991(98)00127-x

[CIT0026] de Vries, H., Kremers, S. P. J., Smeets, T., Brug, J., & Eijmael, K. (2008). The effectiveness of tailored feedback and action plans in an intervention addressing multiple health behaviors. *American Journal of Health Promotion*, *22*(6), 417–425. 18677882 10.4278/ajhp.22.6.417

[CIT0027] Edney, S., Ryan, J. C., Olds, T., Monroe, C., Fraysse, F., Vandelanotte, C., … Maher, C. (2019). User engagement and attrition in an app-based physical activity intervention: Secondary analysis of a randomized controlled trial. *Journal of Medical Internet Research*, *21*(11), e14645. doi:10.2196/14645 31774402 PMC6906621

[CIT0028] Edwards, E. S., & Sackett, S. C. (2016). Psychosocial variables related to why women are less active than men and related health implications. *Clinical Medicine Insights: Women’s Health*, *9s1*(Suppl. 1), 47–56. doi:10.4137/CMWH.S34668 PMC4933535 27398045

[CIT0029] Faul, F., Erdfelder, E., Lang, A.-G., & Buchner, A. (2007). G*Power 3: A flexible statistical power analysis program for the social, behavioral, and biomedical sciences. *Behavior Research Methods*, *39*(2), 175–191. doi:10.3758/bf03193146 17695343

[CIT0030] Friederichs, S. A. H., Oenema, A., Bolman, C., & Lechner, L. (2015). Long term effects of self-determination theory and motivational interviewing in a web-based physical activity intervention: Randomized controlled trial. *International Journal of Behavioral Nutrition and Physical Activity*, *12*(1), 101. doi:10.1186/s12966-015-0262-9 PMC4538737 26283094

[CIT0031] Friederichs, S. A. H., Oenema, A., Bolman, C., & Lechner, L. (2016). Motivational interviewing and self-determination theory in a web-based computer tailored physical activity intervention: A randomized controlled trial. *Psychology & Health*, *31*(8), 907–930. doi:10.1080/08870446.2016.1151018 26849996

[CIT0032] Garriguet, D., & Colley, R. C. (2014). A comparison of self-reported leisure-time physical activity and measured moderate-to-vigorous physical activity in adolescents and adults. *Health Reports*, *25*(7), 3–11. 25029491

[CIT0033] Godin, G., & Shephard, R. J. (1985). A simple method to assess exercise behavior in the community. *Canadian Journal of Applied Sport Sciences*, *10*(3), 141–146. 4053261

[CIT0034] Golsteijn, R. H. J., Bolman, C., Volders, E., Peels, D. A., de Vries, H., & Lechner, L. (2018). Short-term efficacy of a computer-tailored physical activity intervention for prostate and colorectal cancer patients and survivors: A randomized controlled trial. *International Journal of Behavioral Nutrition and Physical Activity*, *15*(1), 106. doi:10.1186/s12966-018-0734-9 PMC6208119 30376857

[CIT0035] Gomez Quiñonez, S., Walthouwer, M. J. L., Schulz, D. N., & de Vries, H. (2016). Mhealth or eHealth? Efficacy, use, and appreciation of a web-based computer-tailored physical activity intervention for Dutch adults: A randomized controlled trial. *Journal of Medical Internet Research*, *18*(11), e278. doi:10.2196/jmir.6171 27829576 PMC5121532

[CIT0036] Gueorguieva, R., & Krystal, J. H. (2004). Move over anova: Progress in analyzing repeated-measures data andits reflection in papers published in the archives of general psychiatry. *Archives of General Psychiatry*, *61*(3), 310–317. doi:10.1001/archpsyc.61.3.310 14993119

[CIT0037] Guthold, R., Stevens, G. A., Riley, L. M., & Bull, F. C. (2018). Worldwide trends in insufficient physical activity from 2001 to 2016: A pooled analysis of 358 population-based surveys with 1·9 million participants. *The Lancet Global Health*, *6*(10), e1077–e1086. doi:10.1016/S2214-109X(18)30357-7 30193830

[CIT0038] Hankonen, N., Absetz, P., Ghisletta, P., Renner, B., & Uutela, A. (2010). Gender differences in social cognitive determinants of exercise adoption. *Psychology & Health*, *25*(1), 55–69. doi:10.1080/08870440902736972 20391207

[CIT0039] Hawkins, R. P., Kreuter, M., Resnicow, K., Fishbein, M., & Dijkstra, A. (2008). Understanding tailoring in communicating about health. *Health Education Research*, *23*(3), 454–466. 18349033 10.1093/her/cyn004 PMC3171505

[CIT0040] Howlett, N., Trivedi, D., Troop, N. A., & Chater, A. M. (2019). Are physical activity interventions for healthy inactive adults effective in promoting behavior change and maintenance, and which behavior change techniques are effective? A systematic review and meta-analysis. *Translational Behavioral Medicine*, *9*, 147–157. doi:10.1093/tbm/iby010 29506209 PMC6305562

[CIT0041] IPAQ. (2005). *International physical activity questionnaire scoring protocol*. Retrieved December 15, 2015, from https://docs.google.com/viewer?a=v&pid=sites&srcid=ZGVmYXVsdGRvbWFpbnx0aGVpcGFxfGd4OjE0NDgxMDk3NDU1YWRlZTM

[CIT0042] Jacobs, D. R., Jr., Ainsworth, B. E., Hartman, T. J., & Leon, A. S. (1993). A simultaneous evaluation of 10 commonly used physical activity questionnaires. *Medicine & Science in Sports & Exercise*, *25*(1), 81–91. 8423759 10.1249/00005768-199301000-00012

[CIT0043] Jahangiry, L., Farhangi, M. A., Shab-Bidar, S., Rezaei, F., & Pashaei, T. (2017). Web-based physical activity interventions: A systematic review and meta-analysis of randomized controlled trials. *Public Health*, *152*, 36–46. doi:10.1016/j.puhe.2017.06.005 28734170

[CIT0044] Kohl, L. F. M., Crutzen, R., & de Vries, N. K. (2013). Online prevention aimed at lifestyle behaviors: A systematic review of reviews. *Journal of Medical Internet Research*, *15*(7), e146. doi:10.2196/jmir.2665 23859884 PMC3714003

[CIT0045] Koivula, N. (1999). Sport participation: Differences in motivation and actual participation due to gender typing. *Journal of Sport Behavior*, *22*(3), 360–380.

[CIT0046] Krebs, P., Prochaska, J. O., & Rossi, J. S. (2010). A meta-analysis of computer-tailored interventions for health behavior change. *Preventive Medicine*, *51*(3-4), 214–221. doi:10.1016/j.ypmed.2010.06.004 20558196 PMC2939185

[CIT0047] LaPlante, C., & Peng, W. (2011). A systematic review of e-Health interventions for physical activity: An analysis of study design, intervention characteristics, and outcomes. *Telemedicine and e-Health*, *17*(7), 509–523. doi:10.1089/tmj.2011.0013 21718092

[CIT0048] Lieber, S. B., Redberg, R. F., Blumenthal, R. S., Gandhi, A., Robb, K. J., & Mora, S. (2012). A national interactive web-based physical activity intervention in women, evaluation of the American heart association choose to move program 2006–2007. *The American Journal of Cardiology*, *109*(12), 1754–1760. doi:10.1016/j.amjcard.2012.02.017 22494850

[CIT0049] Locke, E. A., & Latham, G. P. (1994). Goal setting theory. In H. F. O’Neil, Jr. & M. Drillings (Eds.), *Motivation: Theory and research* (pp. 13–29). Hillsdale, NJ: Lawrence Erlbaum Associates, Inc.

[CIT0050] Lustria, M. L. A., Noar, S. M., Cortese, J., Van Stee, S. K., Glueckauf, R. L., & Lee, J. (2013). A meta-analysis of web-delivered tailored health behavior change interventions. *Journal of Health Communication*, *18*(9), 1039–1069. doi:10.1080/10810730.2013.768727 23750972

[CIT0051] Maher, A. C., Lewis, K. L., Ferrar, K., Marshall, S., De Bourdeaudhuij, I., & Vandelanotte, C. (2014). Are health behavior change interventions that use online social networks effective? A systematic review. *Journal of Medical Internet Research*, *16*(2), e40. doi:10.2196/jmir.2952 24550083 PMC3936265

[CIT0052] McGorrian, C., Yusuf, S., Islam, S., Jung, H., Rangarajan, S., Avezum, A., … Anand, S. S. (2011). Estimating modifiable coronary heart disease risk in multiple regions of the world: The INTERHEART modifiable risk score. *European Heart Journal*, *32*(5), 581–589. doi:10.1093/eurheartj/ehq448 21177699

[CIT0053] Michie, S., Yardley, L., West, R., Patrick, K., & Greaves, F. (2017). Developing and evaluating digital interventions to promote behavior change in health and health care: Recommendations resulting from an international workshop. *Journal of Medical Internet Research*, *19*(6), e232. doi:10.2196/jmir.7126 28663162 PMC5509948

[CIT0054] Molanorouzi, K., Khoo, S., & Morris, T. (2015). Motives for adult participation in physical activity: Type of activity, age, and gender. *BMC Public Health*, *15*, 66. doi:10.1186/s12889-015-1429-7 25637384 PMC4314738

[CIT0055] Moller, A. C., Merchant, G., Conroy, D. E., West, R., Hekler, E., Kugler, K. C., & Michie, S. (2017). Applying and advancing behavior change theories and techniques in the context of a digital health revolution: Proposals for more effectively realizing untapped potential. *Journal of Behavioral Medicine*, *40*(1), 85–98. doi:10.1007/s10865-016-9818-7 28058516 PMC5532801

[CIT0056] Müller, A. M., Maher, C. A., Vandelanotte, C., Hingle, M., Middelweerd, A., Lopez, M. L., … Wark, P. A. (2018). Physical activity, sedentary behavior, and diet-related eHealth and mHealth research: Bibliometric analysis. *Journal of Medical Internet Research*, *20*(4), e122. doi:10.2196/jmir.8954 29669703 PMC5932335

[CIT0057] Neville, L. M., O’Hara, B., & Milat, A. (2009). Computer-tailored physical activity behavior change interventions targeting adults: A systematic review. *International Journal of Behavioral Nutrition and Physical Activity*, *6*, 30. 10.1186/1479-5868-6-30 PMC2700068 19490649

[CIT0058] Noar, S. M., Benac, C. N., & Harris, M. S. (2007). Does tailoring matter? Meta-analytic review of tailored print health behavior change interventions. *Psychological Bulletin*, *133*(4), 673–693. 17592961 10.1037/0033-2909.133.4.673

[CIT0059] Oh, H., Rizo, C., Enkin, M., & Jadad, A. (2005). What is eHealth (3): A systematic review of published definitions. *Journal of Medical Internet Research*, *7*(1), e1. 15829471 10.2196/jmir.7.1.e1 PMC1550636

[CIT0060] Peels, D. A., Hoogenveen, R. R., Feenstra, T. L., Golsteijn, R. H., Bolman, C., Mudde, A. N., … Lechner, L. (2014). Long-term health outcomes and cost-effectiveness of a computer-tailored physical activity intervention among people aged over fifty: Modelling the results of a randomized controlled trial. *BMC Public Health*, *14*, 1099. doi:10.1186/1471-2458-14-1099 25342517 PMC4221676

[CIT0061] Peels, D. A., van Stralen, M. M., Bolman, C., Golsteijn, R. H., de Vries, H., Mudde, A. N., & Lechner, L. (2012). Development of web-based computer-tailored advice to promote physical activity among people older than 50 years. *Journal of Medical Internet Research*, *14*(2), e39. doi:10.2196/jmir.1742 22390878 PMC3376526

[CIT0062] Perski, O., Blandford, A., West, R., & Michie, S. (2017). Conceptualising engagement with digital behaviour change interventions: A systematic review using principles from critical interpretive synthesis. *Translational Behavioral Medicine*, *7*(2), 254–267. doi:10.1007/s13142-016-0453-1 27966189 PMC5526809

[CIT0063] Peters, S. A. E., Bots, M. L., den Ruijter, H. M., Palmer, M. K., Grobbee, D. E., Crouse, J. R., … Koffijberg, H. (2012). Multiple imputation of missing repeated outcome measurements did not add to linear mixed-effects models. *Journal of Clinical Epidemiology*, *65*(6), 686–695. doi:10.1016/j.jclinepi.2011.11.012 22459429

[CIT0064] Peyman, N., Rezai-Rad, M., Tehrani, H., Gholian-Aval, M., Vahedian-Shahroodi, M., & Heidarian Miri, H. (2018). Digital media-based health intervention on the promotion of women’s physical activity: A quasi-experimental study. *BMC Public Health*, *18*(1), 134. doi:10.1186/s12889-018-5025-5 29334970 PMC5769504

[CIT0065] Plaete, J., De Bourdeaudhuij, I., Verloigne, M., & Crombez, G. (2015). Acceptability, feasibility and effectiveness of an eHealth behaviour intervention using self-regulation: ‘MyPlan’. *Patient Education and Counseling*, *98*(12), 1617–1624. doi:10.1016/j.pec.2015.07.014 26277282

[CIT0066] Plotnikoff, R. C., Lubans, D. R., Trinh, L., & Craig, C. L. (2012). A 15-year longitudinal test of the theory of planned behaviour to predict physical activity in a randomized national sample of Canadian adults. *Psychology of Sport and Exercise*, *13*(5), 521–527. doi:10.1016/j.psychsport.2012.02.005

[CIT0067] Prochaska, J. O., & Velicer, W. F. (1997). The transtheoretical model of health behavior change. *American Journal of Health Promotion*, *12*(1), 38–48. 10170434 10.4278/0890-1171-12.1.38

[CIT0068] Reed, J. L., Prince, S. A., Cole, C. A., Nerenberg, K. A., Hiremath, S., Tulloch, H. E., … Reid, R. D. (2015). E-health physical activity interventions and moderate-to-vigorous intensity physical activity levels among working-age women: A systematic review protocol. *Systematic Reviews*, *4*, 3. doi:10.1186/2046-4053-4-3 25589330 PMC4506417

[CIT0069] Reinwand, D. A., Crutzen, R., Elfeddali, I., Schneider, F., Schulz, D. N., Stanczyk, N. E., … de Vries, H. (2015). Impact of educational level on study attrition and evaluation of web-based computer-tailored interventions: Results from seven randomized controlled trials. *Journal of Medical Internet Research*, *17*(10), e228. doi:10.2196/jmir.4941 26446779 PMC4642402

[CIT0070] Reinwand, D. A., Crutzen, R., Storm, V., Wienert, J., Kuhlmann, T., de Vries, H., & Lippke, S. (2016). Generating and predicting high quality action plans to facilitate physical activity and fruit and vegetable consumption: Results from an experimental arm of a randomised controlled trial. *BMC Public Health*, *16*(1), 1–12. doi:10.1186/s12889-016-2975-3 27066779 PMC4828759

[CIT0071] Rhodes, R. E., Blanchard, C. M., Benoit, C., Levy-Milne, R., Jean Naylor, P., Symons Downs, D., & Warburton, D. E. R. (2014). Belief-level markers of physical activity among young adult couples: Comparisons across couples without children and new parents. *Psychology & Health*, *29*(11), 1320–1340. doi:10.1080/08870446.2014.929687 24894608

[CIT0072] Rhodes, R. E., & Dickau, L. (2012). Experimental evidence for the intention–behavior relationship in the physical activity domain: A meta-analysis. *Health Psychology*, *31*(6), 724–727. doi:10.1037/a0027290 22390739

[CIT0073] Romeike, K., Lechner, L., de Vries, H., & Oenema, A. (2016). Development of a computer-tailored nutrition and physical activity intervention for lower-educated women of Dutch, Turkish and Moroccan origin using content matching and ethnic identity tailoring. *BMC Public Health*, *16*(1), 924. doi:10.1186/s12889-016-3596-6 27590408 PMC5010670

[CIT0074] Schneider, F., de Vries, H., Candel, M., van de Kar, A., & van Osch, L. (2013). Periodic email prompts to re-use an internet-delivered computer-tailored lifestyle program: Influence of prompt content and timing. *Journal of Medical Internet Research*, *15*(1), e23. doi:10.2196/jmir.2151 23363466 PMC3636303

[CIT0075] Schulz, D. N., Kremers, S. P. J., Vandelanotte, C., Schneider, F., Candel, M. J., & de Vries, H. (2014). Effects of a web-based tailored multiple-lifestyle intervention for adults: A two-year randomized controlled trial comparing sequential and simultaneous delivery modes. *Journal of Medical Internet Research*, *16*(1), e26. doi:10.2196/jmir.3094 24472854 PMC3936298

[CIT0076] Schulz, D. N., Smit, E. S., Stanczyk, N. E., Kremers, S. P. J., de Vries, H., & Evers, S. M. A. A. (2014). Economic evaluation of a web-based tailored lifestyle intervention for adults: Findings regarding cost-effectiveness and cost-utility from a randomized controlled trial. *Journal of Medical Internet Research*, *16*(3), e91. doi:10.2196/jmir.3159 24650860 PMC3978559

[CIT0077] Shaw, T., McGregor, D., Brunner, M., Keep, M., Janssen, A., & Barnet, S. (2017). What is eHealth (6)? Development of a conceptual model for eHealth: Qualitative study with key informants. *Journal of Medical Internet Research*, *19*(10), e324. doi:10.2196/jmir.8106 29066429 PMC5676031

[CIT0078] Short, C. E., James, E. L., Plotnikoff, R. C., & Girgis, A. (2011). Efficacy of tailored-print interventions to promote physical activity: A systematic review of randomised trials. *International Journal of Behavioral Nutrition and Physical Activity*, *8*, 113. doi:10.1186/1479-5868-8-113 PMC3214130 21999329

[CIT0079] Statistics Canada. (2015). *Directly measured physical activity of adults, 2012 and 2013* (Health fact sheet: Statistics Canada catalogue no. 82-625-X). http://www.statcan.gc.ca/pub/82-625-x/2015001/article/14135-eng.htm

[CIT0080] Storm, V., Dörenkämper, J., Reinwand, D. A., Wienert, J., De Vries, H., & Lippke, S. (2016). Effectiveness of a web-based computer-tailored multiple-lifestyle intervention for people interested in reducing their cardiovascular risk: A randomized controlled trial. *Journal of Medical Internet Research*, *18*(4), e78. doi:10.2196/jmir.5147 27068880 PMC4844907

[CIT0081] Sullivan, T. R., White, I. R., Salter, A. B., Ryan, P., & Lee, K. J. (2018). Should multiple imputation be the method of choice for handling missing data in randomized trials? *Statistical Methods in Medical Research*, *27*(9), 2610–2626. doi:10.1177/0962280216683570 28034175 PMC5393436

[CIT0082] Teo, K., Chow, C. K., Vaz, M., Rangarajan, S., & Yusuf, S. (2009). The prospective urban rural epidemiology (PURE) study: Examining the impact of societal influences on chronic noncommunicable diseases in low-, middle-, and high-income countries. *American Heart Journal*, *158*(1), 1-7.e1. 19540385 10.1016/j.ahj.2009.04.019

[CIT0083] Thabane, L., Mbuagbaw, L., Zhang, S., Samaan, Z., Marcucci, M., Ye, C., … Goldsmith, C. H. (2013). A tutorial on sensitivity analyses in clinical trials: The what, why, when and how. *BMC Medical Research Methodology*, *13*, 92. doi:10.1186/1471-2288-13-92 23855337 PMC3720188

[CIT0084] Twisk, J. W. R. (2006). *Applied multilevel analysis: A practical guide*. Cambridge: Cambridge University Press.

[CIT0085] Twisk, J., de Boer, M., de Vente, W., & Heymans, M. (2013). Multiple imputation of missing values was not necessary before performing a longitudinal mixed-model analysis. *Journal of Clinical Epidemiology*, *66*(9), 1022–1028. doi:10.1016/j.jclinepi.2013.03.017 23790725

[CIT0086] Voncken-Brewster, V., Tange, H., de Vries, H., Nagykaldi, Z., Winkens, B., & van der Weijden, T. (2015). A randomized controlled trial evaluating the effectiveness of a web-based, computer-tailored self-management intervention for people with or at risk for COPD. *International Journal of Chronic Obstructive Pulmonary Disease*, *10*, 1061–1073. doi:10.2147/COPD.S81295 26089656 PMC4467652

[CIT0087] Walthouwer, M. J. L., Oenema, A., Lechner, L., & de Vries, H. (2015). Use and effectiveness of a video- and text-driven web-based computer-tailored intervention: Randomized controlled trial. *Journal of Medical Internet Research*, *17*(9), e222. doi:10.2196/jmir.4496 26408488 PMC4642388

[CIT0088] Watson, N. L., Mull, K. E., Heffner, J. L., McClure, J. B., & Bricker, J. B. (2018). Participant recruitment and retention in remote eHealth intervention trials: Methods and lessons learned from a large randomized controlled trial of two web-based smoking interventions. *Journal of Medical Internet Research*, *20*(8), e10351. doi:10.2196/10351 30143479 PMC6128955

[CIT0089] Webb, T. L., Joseph, J., Yardley, L., & Michie, S. (2010). Using the Internet to promote health behavior change. *Journal of Medical Internet Research*, *12*, e4. doi:10.2196/jmir.1376 20164043 PMC2836773

[CIT0090] Webb, T. L., & Sheeran, P. (2006). Does changing behavioral intentions engender behavior change? A meta-analysis of the experimental evidence. *Psychological Bulletin*, *132*(2), 249–268. 16536643 10.1037/0033-2909.132.2.249

[CIT0091] Xi, W., Pennell, M. L., Andridge, R. R., & Paskett, E. D. (2018). Comparison of intent-to-treat analysis strategies for pre-post studies with loss to follow-up. *Contemporary Clinical Trials Communications*, *11*, 20–29. doi:10.1016/j.conctc.2018.05.008 30023456 PMC6022256

[CIT0092] Yardley, L., Spring, B. J., Riper, H., Morrison, L. G., Crane, D. H., Curtis, K., … Blandford, A. (2016). Understanding and promoting effective engagement with digital behavior change interventions. *American Journal of Preventive Medicine*, *51*(5), 833–842. doi:10.1016/j.amepre.2016.06.015 27745683

